# Anesthetic Related Advances with Cyclodextrins

**DOI:** 10.1100/tsw.2007.83

**Published:** 2007-03-02

**Authors:** Mark Welliver, John P. McDonough

**Affiliations:** University of North Florida, Jacksonville, FL, USA

**Keywords:** cyclodextrins, modified cyclodextrin, encapsulation, inclusion complex, hostguest assembly

## Abstract

Cyclodextrins encapsulate and electrostatically bind to lipophilic molecules. The exterior of cyclodextrins are water-soluble and maintain aqueous solubility despite encapsulation of non-aqueous soluble molecules. This unique ability to encapsulate lipophilic molecules and maintain water solubility confers numerous pharmacologic advantages for both drug delivery and removal. Cyclodextrins, a component part of supramolecular chemistry, may be in its infancy of anesthetic application but recent advances have been described as novel and revolutionary. A review of current research coupled with an understanding of cyclodextrin properties is necessary to fully appreciate the current uses and future potentials of these unique molecules.

## INTRODUCTION

Drug encapsulation technologies are offering many advantages over standard organic solvents to dissolve lipophilic drugs. Typical solvents such as propylene glycol, oil, and benzyl alcohol are toxic and irritating. Aqueous dissolution of drugs would be preferable in most cases if safe effective options existed. Cyclodextrins offer safe solubilization. Cyclodextrins are naturally occurring oligosaccharides consisting of six, seven or eight glucose monomers arranged in a cyclical form created from enzymatic starch conversion (Fig. 1). This cyclical form creates a cone into which another molecule may reside. Unique to these cyclodextrin molecules is their exterior water solubility and interior lipophilicity. Cyclodextrins therefore dissolve easily in water. Lipophilic molecules such as steroids and many aromatic compounds are poorly soluble. Steroids, aromatics and other lipophilic molecules may be encased in a cyclodextrin and subsequently be dissolved in water. Cyclodextrin encapsulation of lipiophilic molecules allows aqueous dissolution (Fig. 2).

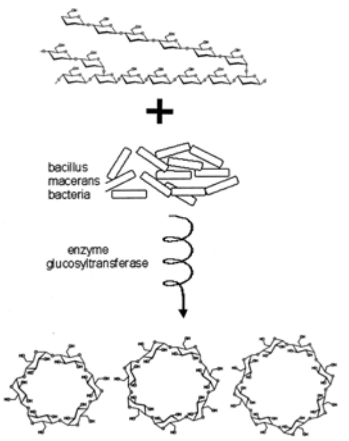

**FIGURE 1.** Enzymatic conversion of starch to natural cyclodextrins. (Reprinted with permission Wacker Chemical Corp. Adrian MI. USA, modified)

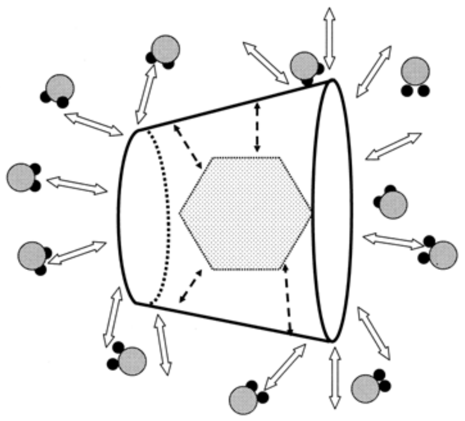

**FIGURE 2.** Lipophilic molecule encapsulated by a cyclodextrin. Note; arrows represent non-covalent thermodynamic attractions.

## THE NATURAL CYCLODEXTRINS

Cyclodextrin discovery originated in the early 1900s but did not become more appreciated until 50 years later when French and Cramer enzymatically produced and purified the natural cyclodextrins[1]. The naturally occurring cyclodextrins are rings of bonded glucose, oligosaccharides, created by the enzyme glucosyltransferase, which is released by Bacillus macerans bacteria. These glucose molecules are bonded together by their first and fourth carbon atoms forming a ring that geometrically resembles a truncated cone (Fig. 3). It is this geometric shape that creates a cavity that allows cyclodextrins to engulf smaller molecules. The natural cyclodextrins are composed of six, seven, or eight glucose molecules and are named alpha, beta and gamma (Fig. 4). The cavity sizes of the alpha, beta, and gamma cyclodextrins are approximately 0.5, 0.6, and 0.8 nanometers respectively[2]. The size of the cavity dictates the size molecule that may be encapsulated. A larger cavity allows a larger molecule to be encapsulated. A gamma cyclodextrin may encapsulate a larger molecule than an alpha cyclodextrin.

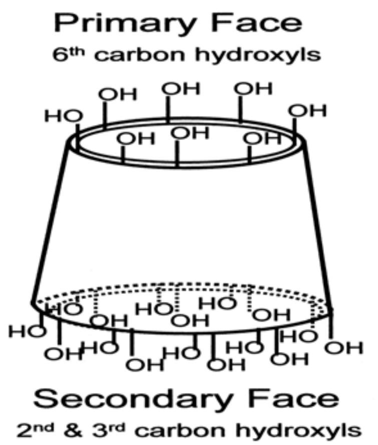

**FIGURE 3.** Truncated cone shape of a gamma cyclodextrin showing location of hydroxyl groups along rims.

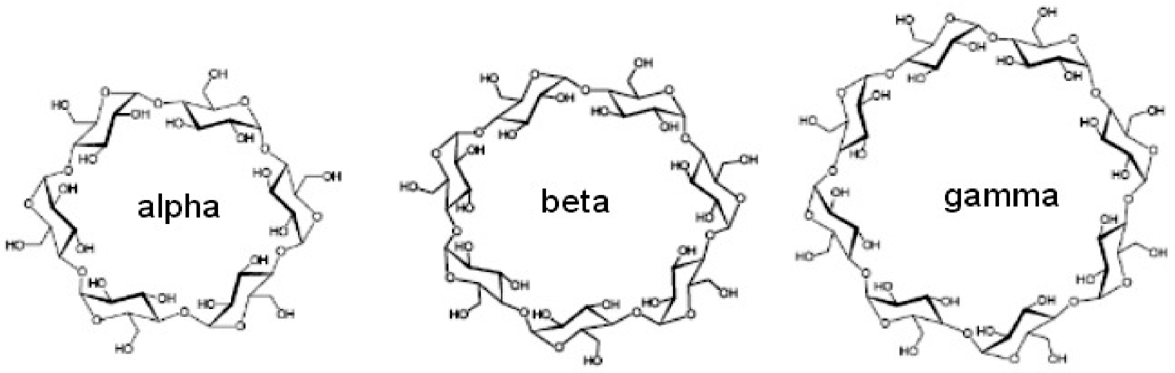

**FIGURE 4.** Naturally occurring cyclodextrins, alpha, beta, and gamma. (Reprinted with permission Wacker Chemical Corp. Adrian MI. USA).

## MODIFIED CYCLODEXTRINS

Modification of the natural cyclodextrins improves the binding affinity for lipophilic drugs and allows targeted encapsulation while limiting encapsulation of other lipophilic molecules. Modified natural cyclodextrins, known as cyclodextrin derivatives, may alter the pharmacokinetics(PK) and pharmacodynamics(PD) of the encased drug. This alteration of PK and PD may improve or lessen the efficacy of the cyclodextrin/ drug formulation. Modifying the natural cyclodextrins often bestows more desirable properties and enables application to drug specific encapsulation. The modification sites available to add substituent groups are the $2^{nd}$, $3^{rd}$, and $6^{th}$ carbon atoms of each glucose unit comprising the cyclodextrin (Figs. 3 & 5). An alpha cyclodextrin composed of six glucose units will therefore have 18 sites for potential modification, a beta cyclodextrin 21, and a gamma 24. Numerous functional groups, ionic or anionic compounds or molecular structures may be added to these sites creating an astronomical number of possible modifications.

Targeted delivery and encapsulation of drugs, improved solubilization, elimination of toxic solvents such as alcohol, propylene glycol, and oil, improved stability and increased shelf life are some of the useful applications of cyclodextrins. These properties imparted by cyclodextrins have generated interest in increasing the safety and efficacy of anesthetic drugs. Multiple anesthetic drug formulations possess undesirable side effects related to the solvent rather than the drug. Replacement of less than optimal solvents with cyclodextrin carriers in an aqueous solution expands the pharmacologic options for drug delivery. Improved pharmacodynamics and safety profiles have also been suggested.

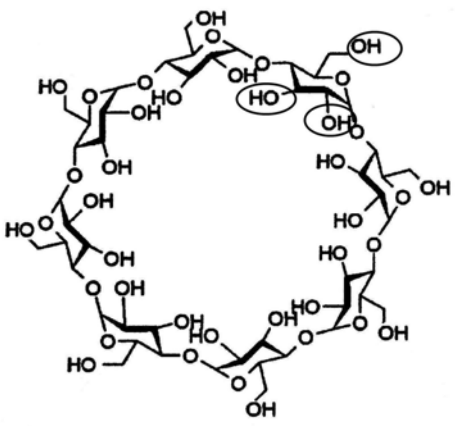

**FIGURE 5.** Modification sites of gamma cyclodextrin on 2nd, 3rd, and 6th carbon (circled). Rreprinted with permission Wacker Chemical Corp. Adrian MI. USA).

## INTRAVENOUS INDUCTION AGENTS

Propofol, a diisopropyl phenol, is not water-soluble and is commercially prepared as a colloidal suspension of oil in water. This preparation has several concerns including pain on injection and the risk of bacterial growth[3]. Numerous studies have attempted to identify and remedy the significant irritation experienced upon injection[4,5,6]. The lipid component of the propofol formulation has been suggested to be the precipitating cause of pain[7]. Pain on injection persists with a new propofol emulsion despite a 50% lower lipid concentration[8]. Reformulation of propofol without a lipid solvent may improve delivery, lessen irritation and potentially decrease the risk of bacterial contamination. Cyclodextrin complexed propofol preparations have been studied with varying results. Viernstein found similar PKs and PDs between a hydroxypropyl-beta-cyclodextrin propofol formulation and a current lipid containing product in rabbit studies[9]. In contrast, Trapani found significant differences in induction times and anesthetic duration with a hydroxypropyl-beta-cyclodextrin formulation compared to a lipid formulation[10]. Bielen also found neuronally mediated cholinergic effects associated with the hydroxypropyl-beta-cyclodextrin formulation that was not found with the lipid formulation[11].

Another propofol formulation using a sulfobutyl ether-beta-cyclodextrin (Captisol®) has shown similar pharmacokinetics and pharmacodynamics to lipid containing propofol preparations[12]. Captisol® is a clear aqueous solution of a modified beta cyclodextrin that has been altered to increase its affinity for propofol yet allow its release upon injection. It has also been shown to possess antimicrobial properties[13]. Continued study of Captisol® enabled propofol is needed to fully elucidate its bioequivalence, stability, toxicity and irritating effects if any. A modified cyclodextrin may offer a promising formulation for an aqueous solution of propofol without the undesirable effects associated with lipid formulations.

Etomidate, a non-barbiturate induction agent, contains a carboxylated imidazole that is lipophilic at physiologic pH and water soluble at acidic pHs. Etomidate is commercially prepared with 35% propylene glycol up to in an acidic aqueous solution that causes significant pain on injection[14]. Propylene glycol has been found to damage vascular smooth muscle and endothelium and cause pain when injected[15,16]. Optimal etomidate preparations would have a neutral pH and would not contain propylene glycol. Doenicke compared a hydroxypropyl-beta-cyclodextrin etomidate formulation containing no propylene glycol to standard propylene glycol containing etomidate and found significant differences[17]. In a study of twenty-four male volunteers receiving either propylene glycol formulated etomidate or cyclodextrin containing formulation, there was a five-fold increase in venous irritation in the propylene glycol group. Three of the five subjects experienced compared with none in the cyclodextrin group. Hemodynamics were similar in both groups. It was concluded that hydroxypropyl-beta-cyclodextrin is superior to propylene glycol as a solvent for etomidate[17]. Additional research is needed, but the findings are encouraging.

Another modified cyclodextrin, a sulfobutyl ether-beta-cyclodextrin, has also been formulated with etomidate. In dogs, this etomidate/ cyclodextrin formulation was found to share similar PKs and PDs with etomidate formulated with propylene glycol. Bioequivalence of this etomidate/ cyclodextrin formulation was demonstrated compared to a standard propylene glycol formulation. Unique to this etomidate/ cyclodextrin formulation was the ability to deliver subcutaneously without apparent irritation. A 12 mg per milliliter aqueous solution of etomidate in 20% sulfobutyl ether-beta-cyclodextrin was well tolerated by the subcutaneous route. These findings led the authors to conclude that a sulfobutyl ether-beta-cyclodextrin enabled etomidate formulation with its lower side effect profile may be a viable alternative to current etomidate preparations[18].

## LOCAL ANESTHETICS

The few studies of local anesthetic cyclodextrin complexes have created interesting possible applications. Cyclodextrins have been shown to improve the aqueous solubility of local anesthetics and decrease precipitate formation when alkalizing these solutions[19]. The analgesic effects of bupivacaine complexed with a hydroxypropyl-beta-cyclodextrin was concluded by de Arauju to be more pronounced than bupivacaine alone[20]. Kopecky sucessfully complexed bupivacaine with both natural and modified cyclodextrins and found the complex readily dissociates at neuronal tissues[21]. Dollo suggested a cyclodextrin bupivacaine complex may improve the therapeutic index as the rate of local anesthetic absorption was slowed but not the quantity[22]. Heavnor found cocaine complexed with a cyclodextrin allowed higher doses to be delivered before rats exhibited toxic effects[23]. This toxicity lowering effect of cyclodextrin-local anesthetic complexes was also found with benzocaine[24]. Though there are few studies of cyclodextrin complexes with local anesthetics, the data suggests that cyclodextrins may offer a safer and more efficacious option for the delivery of local anesthetics. Further study is needed to fully explore these potential benefits.

## BENZODIAZEPINES

Nasal administration is an area of interest for the delivery of high potency drugs that have low bioavailabilty in oral form and for drugs that undergo significant first pass metabolism. The nasal route though is susceptible to irritation and mucosal damage from organic solvents and acidic solutions. Loftsson sucessfully prepared a non-irritating nasal solution of midazolam (17mg per milliliter) complexed with a sulfobutyl ether beta-cyclodextrin. This midazolam-cyclodextrin complex was rapidly absorbed from the nasal cavity reaching maximum concentration at 15 minutes[25]. Gudmundsdottir found the PK and PD effects of nasally delivered midazolam cyclodextrin formulation approached those of intravenous midazolam[26]. Cyclodextrin solubilized benzodiazepines offer novel delivery possibilities.

## OPIOIDS

Intrathecal opioids have been sucessfully utilized though the potential for respiratory depression exists from central spread of the narcotic. Opioids alone or as an adjunct to local anesthetics have been well studied and the investigation of cyclodextrin complexed narcotics may lead to improved intrathecal delivery. Intrathecal sufentanil complexed with a hydroxypropyl-beta-cyclodextrin was found to have an increased duration and analgesic effect. It was postulated that the cyclodextrin complex prevented sufentanil redistribution from the intrathecal space, resulting in the increased effect[27]. Similarly, Jang found prolonged duration of action with morphine, lofentanil, alfentanil, and sufentanil when complexed with a hydroxypropyl-beta-cyclodextrin[28]. It was concluded that cyclodextrins limit the diffusion of opioids from the intrathecal space. In contrast, epidurally delivered opioid cyclodextrin complexes did not show this "slow-release reservoir" action that was found with intrathecally delivered complexed opioids. Bernards studied epidurally delivered opioids: morphine, fentanyl, alfentanil, and sufentanil in both complexed and uncomplexed states. No difference was found between cyclodextrin complexed opioid and uncomplexed opioid diffusion through the spinal meninges[29]. Differences in these findings may suggest a different effect of the cyclodextrin-opioid complexes in the intrathecal space compared that or epidurally delivered cyclodextrin-opioid complexes. The differences may also be related to the *in vivo* model studied by Bernards versus the *in vitro* studies evaluating intrathecal cyclodextrin-opioid complexes. Further study is warranted.

## MUSCLE RELAXANTS

Aminosterioid non-depolarizing muscle relaxants have sucessfully been encapsulated within cyclodextrins. Cyclodextrins, though originally conceived as a solubilizing agent to improve dissolution of rocuronium, when modified were found to possess high affinities for the muscle relaxants rocuronium and vecuronium. One particular modified cyclodextrin, sugammadex, was discovered to exert exceptionally high binding capabilities and this finding changed the focus of study from solubilization of rocuronium to encapsulation for reversal of muscle relaxant effects *in vivo*[30,31].

Sugammadex encapsulates the muscle relaxant, forming a very strong complex that prevents the muscle relaxant from acting at its receptors. The sugammadex-muscle relaxant complex is then excreted mainly by the kidneys[32]. All animal, phase I, and phase II human studies have concluded that sugammadex reversal of rocuronium is complete with a dose-dependant time to recovery averaging less than 3 minutes[33]. Profound neuromuscular block induced by rocuronium has also been successfully reversed[34]. Sugammadex has also been shown to successfully reverse the neuromuscular blocking effects of vecuronium but not the long acting aminosteroid non-depolarzing agent pancuronium in humans[35]. Sugammadex is in phase III study and may be available as early as 2007/2008 pending successful study and regulatory approval. Sugammadex is the first in a new class of drugs known as selective relaxant binding agents (SRBAs). The principle mode of action of SRBAs is encapsulation and non-covalent binding of aminosterioid non-depolarizing muscle relaxants followed by excretion.

## CONCLUSION

Improved solubilization of poorly aqueous drugs by cyclodextrin encapsulation is a novel approach to an ongoing pharmacolgic difficulty. Numerous studies have highlighted benefits imparted by cyclodextrin encapsulation such as improved solubilization and chelation activity. Continual research and evaluation of cyclodextrin-encapsulated drugs may offer safer more effective drug delivery and extraction. These early experiments with anesthetic drugs have highlighted some of the potential benefits of cyclodextrin formulation. Sugammadex and Captisol® enabled propofol are two examples of unique though different applications of cyclodextrins. These as well as other drug preparations are likely to improve drug delivery in the future. Cyclodextrins as carrier agents and binding agents are likely to enter the anesthetic armamentarium and anesthesia provider knowledge of cyclodextrin properties will be essential.
